# Supplementary material for: The association between parental resilience and emotional/behavioural problems in children with autism spectrum disorders: The mediating role of parenting style
Source: PLoS One. 2026 Feb 5;21(2):e0329989. doi: 10.1371/journal.pone.0329989 (PMC12875478; doi:10.1371/journal.pone.0329989)
Supplement: S1 File — (DOCX) [file pone.0329989.s001.docx]

**Survey Questionnaire on Children with Autism Spectrum Disorders and Their Families**

Dear Parent,

Hello! Thank you very much for participating in this survey. I am a graduate student in the Department of Psychology at Northwest Normal University, and this questionnaire is part of my thesis research. The purpose of this survey is to understand the social-emotional abilities of children with autism spectrum disorders and their family upbringing environment. Please fill out the questionnaire based on your recent situation. The survey is for research purposes only and does not involve personal information. Your responses will be kept confidential. Thank you very much for your support!

**I. Basic Family Information**

You are the child’s:

A. Mother

B. Father

Father’s age:

A. 20-30 years old

B. 30-40 years old

C. Over 40 years old

Mother’s age:

A. 20-30 years old

B. 30-40 years old

C. Over 40 years old

Father’s education level:

A. Junior high school or below

B. High school or vocational school

C. University

D. Postgraduate or above

Mother’s education level:

A. Junior high school or below

B. High school or vocational school

C. University

D. Postgraduate or above

**II. Basic Information About the Child**

Child’s gender:

A. Male

B. Female

Date of birth: _________ Year _________ Month

Date of diagnosis: _________ Year _________ Month

Diagnosis received at the hospital (multiple choices allowed):

A. Mild Autism

B. Moderate Autism

C. Severe Autism

**III. Strengths and Difficulties Questionnaire (SDQ) Parent Version**

For each of the following questions, please check the box that best describes your child’s situation. Please base your answers on your child’s behavior over the past six months. Be sure to answer every question, even if you are not entirely certain or familiar with a particular issue.

| No. | Item | Not True | Somewhat True | Certainly True |

|-----|------|-----------|---------------|----------------|

| 1 | Considers the feelings of others |   |   |   |

| 2 | Restless, overactive, cannot stay still for long |   |   |   |

| 3 | Often complains of headaches, stomachaches, or sickness |   |   |   |

| 4 | Shares readily with other children (e.g., toys, treats, pencils) |   |   |   |

| 5 | Often has temper tantrums or hot tempers |   |   |   |

| 6 | Rather solitary, tends to play alone |   |   |   |

| 7 | Generally obedient, usually does what adults request |   |   |   |

| 8 | Has many worries, often seems worried |   |   |   |

| 9 | Helpful if someone is hurt, upset, or feeling ill |   |   |   |

| 10 | Constantly fidgeting or squirming |   |   |   |

| 11 | Has at least one good friend |   |   |   |

| 12 | Often fights with other children or bullies them |   |   |   |

| 13 | Often unhappy, depressed, or tearful |   |   |   |

| 14 | Generally liked by other children |   |   |   |

| 15 | Easily distracted, concentration wanders |   |   |   |

| 16 | Nervous or clingy in new situations, easily loses confidence |   |   |   |

| 17 | Kind to younger children |   |   |   |

| 18 | Often lies or cheats |   |   |   |

| 19 | Picked on or bullied by other children |   |   |   |

| 20 | Often volunteers to help others (parents, teachers, other children) |   |   |   |

| 21 | Thinks things out before acting |   |   |   |

| 22 | Steals from home, school, or elsewhere |   |   |   |

| 23 | Gets along better with adults than with other children |   |   |   |

| 24 | Many fears, easily scared |   |   |   |

| 25 | Sees tasks through to the end, good attention span |   |   |   |

**IV Connor-Davidson Resilience Scale (CD-RISC) for Parents**

The following is a self-rating scale used to assess psychological resilience levels. Please indicate the degree to which each statement below applies to you over the past month. Please note that there are no right or wrong answers to these questions.

Item Never Rarely Sometimes Often Always

1. I can adapt to change 0 1 2 3 4

2. I have close, secure relationships 0 1 2 3 4

3. Sometimes, fate or God can help 0 1 2 3 4

4. No matter what happens, I can handle it 0 1 2 3 4

5. Past successes give me confidence to face challenges 0 1 2 3 4

6. I can see the humorous side of things 0 1 2 3 4

7. Coping with stress makes me feel stronger 0 1 2 3 4

8. After hardship or illness, I tend to recover quickly 0 1 2 3 4

9. Things happen for a reason 0 1 2 3 4

10. No matter the outcome, I always try my best 0 1 2 3 4

11. I can achieve my goals 0 1 2 3 4

12. I don’t give up easily when things seem hopeless 0 1 2 3 4

13. I know where to seek help 0 1 2 3 4

14. Under pressure, I can stay focused and think clearly 0 1 2 3 4

15. I like to take the lead in solving problems 0 1 2 3 4

16. I don’t get discouraged by failure 0 1 2 3 4

17. I see myself as a strong person 0 1 2 3 4

18. I can make unusual or difficult decisions 0 1 2 3 4

19. I can deal with unpleasant emotions 0 1 2 3 4

20. I have to act on my intuition 0 1 2 3 4

21. I have a strong sense of purpose 0 1 2 3 4

22. I feel in control of my life 0 1 2 3 4

23. I enjoy challenges 0 1 2 3 4

24. I work hard to achieve my goals 0 1 2 3 4

25. I take pride in my achievements 0 1 2 3 4

**V **Parenting Styles and Dimensions Questionnaire (PSDQ)****

Below are some statements regarding parenting methods. Please rate how frequently you would engage in each of the following behaviors based on your own situation. Consider carefully and respond accordingly.

Item Never=1 Rarely=2 Sometimes=3 Usually=4 Always=5

1 I respond to my child’s feelings and needs. 1 2 3 4 5

2 I use physical punishment to discipline my child. 1 2 3 4 5

3 I take my child’s opinion into account before asking them to do things. 1 2 3 4 5

4 When my child asks why they must obey, I answer: “Because I said so.” 1 2 3 4 5

5 I explain to my child how we feel about their good or bad behaviour. 1 2 3 4 5

6 I spank my child when they are disobedient. 1 2 3 4 5

7 I encourage my child to talk about what bothers them. 1 2 3 4 5

8 I find it hard to discipline my child. 1 2 3 4 5

9 Even when we disagree, I encourage my child to express their views. 1 2 3 4 5

10 I take away privileges without giving adequate explanations as a punishment. 1 2 3 4 5

11 I emphasise the reasons behind rules. 1 2 3 4 5

12 I offer comfort and understanding when my child is upset. 1 2 3 4 5

13 I yell at my child when they misbehave. 1 2 3 4 5

14 I praise my child when they behave well. 1 2 3 4 5

15 I give in to my child when they fuss about something. 1 2 3 4 5

16 I explode in anger toward my child. 1 2 3 4 5

17 I threaten punishment more often than I actually use it. 1 2 3 4 5

18 When making family plans, I take my child’s preferences into account. 1 2 3 4 5

19 I grab my child roughly when they are disobedient. 1 2 3 4 5

20 I threaten punishment but do not actually carry it out. 1 2 3 4 5

21 I encourage my child to express opinions to show respect for their views. 1 2 3 4 5

22 I let my child give input into family rules. 1 2 3 4 5

23 I scold or criticise my child to help them improve. 1 2 3 4 5

24 I spoil my child. 1 2 3 4 5

25 I explain why rules must be obeyed. 1 2 3 4 5

26 I threaten punishment without adequate reason. 1 2 3 4 5

27 I share warm, intimate moments with my child. 1 2 3 4 5

28 I delay letting my child do things as a punishment without adequate reason. 1 2 3 4 5

29 I encourage my child to discuss the consequences of their behaviour. 1 2 3 4 5

30 I scold or criticise my child when they fail to meet my expectations. 1 2 3 4 5

31 I explain the consequences of my child’s behaviour. 1 2 3 4 5

32 I slap my child when they misbehave. 1 2 3 4 5

****Ⅵ Scoring Method for Each Variable****

1. Parenting Styles and Dimensions Questionnaire (PSDQ)

Parenting Style Sub-dimension Code Item numbers

Authoritative Reasoning/Induction Cyd 5 11 25 29 31

Authoritative Warmth/Support Cwn 1 7 12 14 27

Authoritative Autonomy Granting Czz 3 9 18 21 22

Authoritarian Irrational Control Ccf 4 10 26 28

Authoritarian Physical Coercion Cap 2 6 19 32

Authoritarian Verbal Hostility Cyy 13 16 23 30

Permissive Laissez-faire CfzO 8 15 17 20 24

2. Connor-Davidson Resilience Scale (CD-RISC, 25 items, Likert 0-4)

Dimension Item numbers Code Score range

Tenacity (坚韧性) 11 12 13 14 15 16 17 18 19 20 21 22 23 Bjr 0-4

Strength (力量性) 1 5 7 8 9 10 24 25 Bll 0-4

Optimism (乐观性) 2 3 4 6 Blg 0-4

Total Score Bzf 0-100 (sum of Bjr+Bll+Blg)

Interpretation <60 poor 61-69 fair 70-79 good ≥80 excellent

3. Strengths and Difficulties Questionnaire – Parent Report (SDQ-Parent, 25 items, Likert 0-1-2)

Subscale Item numbers Reverse items Scoring

Emotional Symptoms (Cqx) 3 8 13 16 24 none 0-2

Conduct Problems (Cpx) 5 7 12 18 22 7 18 0-2

Hyperactivity (Cdd) 2 10 15 21 25 21 25 0-2

Peer Problems (Ctb) 6 11 14 19 23 11 14 0-2

Total Difficulties (Ckn) Cqx+Cpx+Cdd+Ctb none 0-40

Pro-social Behaviour (Cqsh) 1 4 9 17 20 none 0-2
