# Supplementary material for: The association between parental resilience and emotional/behavioural problems in children with autism spectrum disorders: The mediating role of parenting style
Source: PLoS One. 2026 Feb 5;21(2):e0329989. doi: 10.1371/journal.pone.0329989 (PMC12875478; doi:10.1371/journal.pone.0329989)
Supplement: Attachment — Submitted filename: ASD Parental resilience.pdf [file pone.0329989.s004.pdf]

The article is well-written and addresses a very timely and interesting topic: the parental role in families with a child with Autism Spectrum Disorder.

The authors' aim was to demonstrate how high parental resilience can improve children's internalizing and externalizing symptoms through the adoption of an authoritative parenting style. The English is fluent and easily understandable. The introduction is well-written and comprehensive. A strengths is the employment of standardized and validated neuropsychological scales.

In my opinion, there are some methodological issues that should be addressed by the authors who request minor revisions:

1) METHOD:
The main methodological issues, in my opinion, concern sample selection, inclusion/exclusion criteria, the Autism Diagnosis, and the assessment of autism level.
My questions are the following: a) Who made the autism diagnosis? (Please specify whether it was from a level II center with a medical and psychological team, and whether neuropsychometric tests were performed to support it, or instrumental or genetic investigations to exclude it.)
b) What criteria were used to make the diagnosis? (Please specify whether it was DSM-5.)
c) Given the extreme heterogeneity of autism (we now speak of an autism spectrum and subtypes), was an assessment of the autism level performed? (Please specify whether participants were Level I, II, or II; if this information is not available, it should be included in the study limitations, as the level of autism severity could be a factor influencing parenting stress, resilience, and the presence of internalizing and externalizing problems.)
- What were the exclusion/inclusion criteria? Were neurological and psychiatric comorbidities and relevant clinical conditions excluded? These factors could also influence the above-mentioned aspects.

Minor issues:
- The age range is quite broad. Did the authors perform a data analysis dividing the sample into children and adolescents?
This point could be further explored or included as a weakness.
- The questionnaires were completed by 1/5 of fathers and 4/5 of mothers. How do you interpret this data? Is there a difference in resilience, parenting style, and SDQ scores based on the parent completing the questionnaire?
- Was the parents' social background and educational level analyzed?
- Is there information available indicating whether the parents were receiving parenting support?

2) DISCUSSION:
The authors state that greater parental resilience predicts better scores in children with ASD on internalizing and externalizing symptoms, however, this relationship may also be <u>bidirectional.</u>
Numerous articles in the literature report higher parental stress in children with ASD and other neurodevelopmental disorders compared to typically developing children, and parental stress may also be linked to child factors such as the severity of autism symptoms, age, and, above all, general adaptive functioning and emotional-behavioral problems (see *Operto FF et al. Neuropsychological Profile, Emotional/Behavioral Problems, and Parental Stress in Children with Neurodevelopmental Disorders. Brain Sci. 2021 Apr 30;11(5):584. doi: 10.3390/brainsci11050584; Operto FF et al. Adaptive Behavior, Emotional/Behavioral Problems and Parental Stress in Children With Autism Spectrum Disorder. Front Neurosci. 2021 Nov 25;15:751465. doi: 10.3389/fnins.2021.751465.*).
This point should be carefully addressed in the discussion. More problematic or more challenging children may place a strain on their parents over time, resulting in increased parenting stress and

subsequently lower resilience, with dysfunctional consequences for the entire family well being. Furthermore, we say that parenting stress in chronic situations may increase over time.
The "Limitations and Future Perspectives" section should be expanded to include the aforementioned limitations of the study. For future perspectives, it would be interesting to propose a longitudinal study on evolutionary trajectories over time.
