# Supplementary material for: The association between parental resilience and emotional/behavioural problems in children with autism spectrum disorders: The mediating role of parenting style
Source: PLoS One. 2026 Feb 5;21(2):e0329989. doi: 10.1371/journal.pone.0329989 (PMC12875478; doi:10.1371/journal.pone.0329989)
Supplement: Attachment — Submitted filename: Response to Reviewers.docx [file pone.0329989.s003.docx]

Dear Editor,

I sincerely appreciate your response and have carefully implemented all requested revisions per your journal’s guidelines. The modifications are summarized below:

**First**, I reviewed the ethics checklist, responding to each item point-by-point. Additionally, I supplemented critical information at two locations in the Methods section:

- Lines 177-181:"In this study, a web-based questionnaire was used to collect data. An online survey was conducted from June 17, 2024 to April 24, 2025. All participants filled out an informed consent form before the survey began. The study was approved by the Ethics Committee of the Northwest Normal University, China, Approval Number: 2024167, dated:16 May 2024, for studies involving humans. "
- Lines 247-250:"This retrospective study utilized survey data, accessed for research purposes on 29 May 2025. At no point during data extraction, analysis, or reporting did the research team have access to personally identifiable information about study participants. All data were analyzed in anonymized form."

**Second**, I relocated the table from the main text to a separately uploaded TIFF file.

**Finally**, I integrated the title page content into the manuscript’s first page and removed the standalone title page file.

These revisions fully address the editorial requirements. I deeply value the opportunity to publish in your prestigious journal and thank you for your time and consideration. Best regards for your work and well-being.

Best regards,

Jing zhao
