# Supplementary material for: The association between parental resilience and emotional/behavioural problems in children with autism spectrum disorders: The mediating role of parenting style
Source: PLoS One. 2026 Feb 5;21(2):e0329989. doi: 10.1371/journal.pone.0329989 (PMC12875478; doi:10.1371/journal.pone.0329989)
Supplement: Attachment — Submitted filename: Response_to_Reviewers_auresp_1.docx [file pone.0329989.s005.docx]

Dear editor and reviewers,

We now resubmit our manuscript after substantial revision. We thank the reviewers for their excellent comments, which have helped tremendously to improve the quality of the paper. Attached are the point-by-point responses to the reviewer comments, the revised version of our manuscript (with changes highlighted in blue) and a clean version.

We hope that the revised version of this manuscript can resolve your concerns. We would be glad to respond to any further questions and comments.

Below, reviewer's comments and our responses are presented in **black** and **blue**, respectively.

**Editorial Office’s Comments:**

1. Consider changing the title to association. Don’t use effects.

**Reply**: Thank you for your valuable suggestion. We have carefully considered your feedback and revised the term "effects" in the title to "association" to enhance precision in our wording. Additionally, the title format has been adjusted to comply with the journal’s guidelines. The revised title now reads: "The association between Parental Resilience and Emotional/Behavioural Problems in children with Autism Spectrum Disorders: The mediating role of Parenting Style." Thank you once again for your careful review.

2. Abstract: Avoid causative terms, e.g., influence

**Reply:** Thank you for your insightful comment. We have thoroughly reviewed the entire abstract and replaced all vocabulary that might imply causal relationships (such as "influence") with more objective and neutral terms, such as "association".

3. Abstract:Add age range of children with ASD in the abstract

**Reply:** Thank you for your reminder. We have clarified the age range of the study participants (aged 3-18 years) in the abstract.

4. Background: The background is too length, and can you revise and make it concise as there is no concurrent flow when I read and I am losing the true information. Try to keep it to 1.5 pages max. I would suggest revising the background accordingly. Background about ASD, then introduce about Social emotional problems, then resilience and few theories.

**Reply:** Thank you very much for your constructive guidance. We fully agree with your perspective. We have accordingly undertaken a substantial rewrite and condensation of the Introduction section. Following the logical sequence you recommended, we have thoroughly restructured the Introduction. The revised version has been significantly shortened from the original 6 pages to 2.5 pages (530 words, which corresponds to 1.5 pages with single spacing). We would like to reiterate our sincere appreciation for your patience and professional guidance throughout the review process.

5. Line 36: remove serious, Add the current prevalence of ASD

**Reply:** We extend our gratitude for your professional advice, which has significantly enhanced the completeness of the introduction section. In accordance with your suggestion, we have removed the term "severity" from line 36 and supplemented the current epidemiological incidence data for ASD. The added sentence reads: "The prevalence of ASD in China between 2017 and 2023 was 7 per 1,000, indicating an increasing trend compared to the rate reported before 2017 (26.50 per 10,000)" (see lines 48-49).

6. There is no bridge between line 56 and below, can authors write a connection or sentence to act as link.

**Reply:** Thank you for pointing out this issue with the logical flow. We fully agree with your observation. To address this, we have added a transitional sentence at the end of the first paragraph. For your convenience, the added content is provided below:

"Consequently, there is a need to identify and strengthen the internal psychological resources that enable parents to cope effectively. Psychological resilience has been identified by contemporary research as a critical psychological resource (see lines 57-60)."

This sentence is intended to summarize the preceding discussion on interventions for emotional and behavioral problems in children with ASD and to smoothly transition to the subsequent focus on psychological resilience. We sincerely appreciate your constructive feedback on the manuscript's structure, which has provided us with valuable insights for improving the writing.

7. Why do you keep subsection under background – remove. Follow the guidelines.

**Reply:** We sincerely apologize for the formatting oversight. In strict accordance with the journal’s guidelines, we have removed all subsections under the "Background" heading and integrated their content into a coherent paragraph. Thank you once again for your reminder and guidance.

8. Check for formatting. Why does author often provide hyphen in the text.

**Reply:** I sincerely apologize for this formatting error. We have thoroughly reviewed the entire manuscript to ensure full compliance with the journal's formatting requirements, standardized the use of hyphens throughout the text, and replaced em dashes with appositives or attributive clauses to enhance readability and flow. Thank you once again for your invaluable suggestions.

9. Line 72: Yes, agreed. However, you need to briefly touch base on negative effects of being resilience.

**Reply:** Thank you for your valuable suggestion. In strict compliance with your guidance, we have removed the reference to "Empirical evidence" to enhance the rigor of our discussion. Regarding the potential negative effects of psychological resilience, we have supplemented the relevant content in lines 63-65 of the original text. We sincerely appreciate your professional guidance throughout the review process.

10. Line 107: What do you mean by cognitive neuroscience findings.

**Reply:** We sincerely thank the reviewer for this valuable comment. We acknowledge that the original phrase "cognitive neuroscience findings" was indeed insufficiently precise. After careful consideration, and given that the theoretical framework and interpretations of this study are grounded solely at the behavioral level without involving cognitive neural mechanisms, we determined that introducing specific neuroscience research at this point would not be the most appropriate approach. This decision also aligns with the goal of streamlining the Introduction section. Consequently, we have removed the ambiguous expression in line 107 to sharpen the focus of the manuscript. We kindly invite you to review this modification.

11. I can’t find any research-gap (Why was it placed in hypothesis), and there is no details of what previous people have shown using this.

**Reply:** Thank you for your important feedback. We have revised the Introduction section accordingly. First, we have supplemented the literature review on the relationships among the three core variables: "parental psychological resilience," "parenting styles," and "children's emotional and behavioral problems." Building on this, we further articulated a potential mediating pathway—"parental psychological resilience → parenting styles → children's emotional and behavioral problems"—thereby providing a solid logical foundation for the research hypotheses. We sincerely appreciate your professional guidance once again.

12. Line 170: Can you make it clear it is parents’ age. If its mother or father, please specify

**Reply:** Thank you for your reminder. To protect the privacy of children with autism spectrum disorder and in line with established research practices in this field (Gao & Drani, 2024), we only collected age range information for the children. Regarding the demographic variables you highlighted, we have now supplemented the "Methods - Participants" section with detailed parental age data. The specific additions are as follows: "Among the fathers, 6 (2.3 %) were 20-30 years old, 113 (43.8 %) were 30-40, and 139 (53.9 %) were over 40 (see lines 109-110). Among mothers, 6 (2.3 %) were aged 20-30, 131 (50.8 %) were 30-40, and 121 (46.9 %) were over 40 (see lines 112-113)." Thank you once again for your careful proofreading.

(Reference: Gao, X., & Drani, S. (2024). Parent-implemented interventions in Chinese families of children with autism spectrum disorder. Humanities and Social Sciences Communications, 11(1), 1-8.)

13. Line 230: Who were involved in administering and collection details, any authors?

**Reply:** Thank you for raising this question. The specific details regarding data collection and management in this study are as follows:

"The entire research process, including the data management plan, was reviewed, coordinated, and comprehensively supervised by the Ethics Committee of the School of Psychology at Northwest Normal University, ensuring full compliance with ethical standards and the research protocol. During data collection, all questionnaires were administered by systematically trained psychology graduate students (see lines 124-125)."

14. Can you add more details of the aims with stats in data analysis section. For e.g., in results authors are conducting EFA…bivariate analysis and so on…nothing is described.

**Reply:** Thank you for raising this issue. We have supplemented the "Data Analysis" section with comprehensive details as follows:

"This study utilized SPSS 26.0 and Mplus 8.3 for data processing and analysis. Firstly, descriptive statistics and correlation analyses were conducted in SPSS 26.0; Secondly, independent samples *t*-tests were performed in SPSS 26.0 to examine whether there were significant differences in the variables based on parental gender and child gender; Finally, structural equation modeling was conducted in Mplus 8.3 to explore the relationship between parental psychological resilience and emotional/behavioral problems in children with autism, as well as the mediating role of parenting styles in this relationship. The Bootstrap method was employed to test the significance of the effect sizes(see lines 175-183)."

Thank you once again for your valuable suggestions, which have significantly contributed to improving the structure of our manuscript.

15. I would strongly recommend adding all the tests run by the authors to be presented in this section.

**Reply:** Thank you for reiterating this point, which is crucial for enhancing the rigor and clarity of the Methods section. We fully understand your concern and have thoroughly revised and improved the "Data Analysis" subsection in the revised manuscript. To facilitate your review, the modified content is presented below:

"This study utilized SPSS 26.0 and Mplus 8.3 for data processing and analysis. First, descriptive statistics and correlation analyses were conducted in SPSS 26.0; Next, independent samples t-tests were performed in SPSS 26.0 to examine whether there were significant differences in the variables based on parental gender and child gender; Finally, structural equation modeling was conducted in Mplus 8.3 to explore the relationship between parental psychological resilience and emotional/behavioral problems in children with autism, as well as the mediating role of parenting styles in this relationship. The Bootstrap method was employed to test the significance of the effect sizes.

The structural equation modeling analysis was conducted using the maximum likelihood method. Model goodness-of-fit was evaluated using the chi-square/degrees of freedom ratio (χ²/df), comparative fit index (CFI), Tucker-Lewis index (TLI), root mean square error of approximation (RMSEA), and standardized root mean square residual (SRMR). Based on a comprehensive comparison of fit indices by researchers [27], the following criteria were adopted in this study for an acceptable model fit: χ²/df generally less than 5, CFI and TLI greater than 0.90, and RMSEA and SRMR less than 0.08(see lines 175-196)."

Thank you once again for your patient review and valuable guidance.

16. Whether all the variables are normally distributed?

**Reply:** Thank you for raising this point. We have calculated the skewness and kurtosis for all variables. The absolute values of skewness for all variables were ≤ 2, and the absolute values of kurtosis were ≤ 4. This range is methodologically considered acceptable (Kline, 2016), indicating the absence of severe non-normality in the data.

The relevant content has been added to the manuscript (lines 184-189). For your convenience during review, the supplemented text is presented below:"In this study, the absolute values of skewness for all variables were less than 2, and the absolute values of kurtosis were less than 4. According to the standard proposed by Kline (2016)[28], if the absolute value of the skewness coefficient is less than 3 and the absolute value of the kurtosis coefficient is less than 10, the variables are considered to follow or approximately follow a normal distribution. Thus, the data did not exhibit severe non-normality."

Thank you again for your careful assessment, which guided a deeper evaluation and discussion of the data distribution, substantially strengthening the rigor of our analytical approach.

(References: Kline RB. Principles and practice of structural equation modeling, (pp. xvii, 534). Guilford Press; 2016.)

17. Provide details of IQ of these children, how many had comorbid，Add details of familial history of mental illness

**Reply:** Thank you for raising this important issue. We fully agree that these variables are crucial for comprehensively characterizing the sample. Below, we provide clarifications regarding the three points mentioned:

First, regarding comorbid conditions in children, we have supplemented the "Participants" section in the revised manuscript. We apologize for any misunderstanding caused by the lack of clarity in our original description. For your convenience, the added content is as follows: "Parents reported no other serious comorbid conditions (see line 123)."

Second, concerning children's IQ data, we initially planned to assess this during the research design phase. However, we encountered methodological challenges during actual data collection. Many didn’t complete the standardized intelligence tests.

Finally, regarding family history of mental illness, collecting such information involves highly sensitive familial privacy. Based on strict ethical considerations and under the guidance of the ethics committee, we ultimately decided not to systematically collect this type of information in the current study to avoid imposing unnecessary psychological burden or privacy risks on participants.

We sincerely acknowledge that the incomplete coverage of IQ and family psychiatric history constitutes a limitation of this study, and we will certainly prioritize such issues in future research. We appreciate your rigorous review once again.

18. How were these children diagnosed, who diagnosed and criteria?

**Reply:** Thank you for highlighting this crucial point. We have now explicitly supplemented the diagnostic criteria in the "Participants" subsection of the "Methods" section. The specific addition reads:

"All children with ASD were diagnosed by child psychiatrists from tertiary hospitals with extensive experience in autism diagnosis, following the diagnostic criteria outlined in the Diagnostic and Statistical Manual of Mental Disorders, Fourth or Fifth Edition (DSM-IV/DSM-5) (see lines 116-119)." Since the DSM-5 was published in 2013, and our study included children across a wide age range, the use of DSM-IV for some older children (diagnosed earlier) reflects the actual transitional phase in clinical practice.

We sincerely appreciate your valuable feedback once again.

19. Add SEM figure.

**Reply:** Thank you for your reminder. We have added the complete structural equation modeling (SEM) diagram as Figure 2 in the revised manuscript. This figure clearly displays all the latent variables, observed variables, and the standardized path coefficients (in order to maintain a clear and concise presentation, non-significant paths were omitted from the model diagram.) tested in the study.

Figure 2 (Fig.2.tif) is a TIFF file compressed with LZW, 300 dpi in both directions, 1 931 × 1 371 pixels, 8-bit grayscale, ≈1.3 MB, which meets all PLOS ONE requirements (300–600 dpi; 789–2 250 px width and ≤ 2 625 px height at 300 dpi; < 10 MB); fonts are 12 pt Times New Roman and the caption is provided in the manuscript text, not within the image.

For your convenience during the review process, we present the SEM diagram below:

**Fig 2. Structural equation model(N=258).** **p*< 0.05，***p*< 0.01, ****p*< 0.001，parameter estimates were based on 5,000 bootstrap samples; all coefficients in the figure are standardized estimates. The model specified residual correlation between permissive and authoritarian parenting styles. Parental education levels, child age, and autism severity were included as control variables. For simplicity, only significant paths are labeled. Dashed lines for residual correlations of dependent variables indicate non-significance, while solid lines indicate significance.

20. Line 286: Independent t-test between whom? Nothing is clear. How come 80-20% in sex won’t show any differences. I am bit concerned. I would like to see the results of these data as Tables.

**Reply:** We appreciate your feedback. In response, we have added a dedicated subsection for independent samples *t*-tests in the "Results" section. Following your suggestion, the findings regarding group differences based on parent gender and child gender have been organized into tables (see Table 2 at line 235 and Table 3 at line 243). These tables clearly present the comparison groups, means, standard deviations, and *t*-values.

Regarding your concern about the lack of significant differences despite the gender imbalance (approximately 80% mothers vs. 20% fathers), our analyses did identify a significant difference in parental education level. For this reason, we included both fathers' and mothers' education levels as separate control variables in the model. Furthermore, a significant age difference was found between boys and girls with ASD. Consequently, child age was also incorporated into the structural equation model. All relevant statistical results have been fully reported.

21. I would strongly recommend considering Sex in the model, or a sub-group analysis, particularly for male.

**Reply:** We fully concur with the reviewer's insightful perspective. In accordance with your recommendation, we conducted subgroup structural equation modeling analyses separately for female (n=210) and male (n=48) caregiver groups. The analytical results are as follows:

a) In the female caregiver group, the hypothesized mediating model of "psychological resilience → parenting style → children's emotional and behavioral problems" was well-supported by the data, with statistically significant path coefficients and satisfactory model fit indices (χ²(115) = 219.443, χ²/df = 1.91, SRMR = .064, RMSEA = .066 (90% CI: .052–.079), CFI = .936, TLI = .908). The structural equation modeling (SEM) diagram is shown below.

b) In the male caregiver group, the same model demonstrated a poor fit (χ²(115) = 205.949, χ²/df = 1.79, SRMR = .155, RMSEA = .128 (90% CI: .100–.156), CFI = .798, TLI = .710). We primarily attribute this to the small sample size of this subgroup (n=48).

Additionally, we supplemented independent samples t-tests for all variables across parent gender and child gender. The results indicated that, for parent gender, no significant differences were found in any variables except for parental education level. Regarding child gender, no significant differences were observed in any variables except for child age. These findings suggest that fathers and mothers, as well as autistic boys and girls, do not exhibit substantial differences across the study variables. Accordingly, in the subsequent structural equation modeling, we included father's education level, mother's education level, and child gender as control variables.

We extend our sincere gratitude for this highly valuable suggestion, which has significantly enhanced the depth and completeness of our research analysis.

22. Can authors look childhood vs adolescence like a sub-group, strongly recommend to do subgroup analysis.

**Reply:** We sincerely appreciate this important suggestion. Following your recommendation, we performed additional age-based subgroup analyses by dividing the sample into a child group (3–12 years, n = 194) and an adolescent group (13–18 years, n = 64). The results are as follows:

a) In the child group, the hypothesized theoretical model of "psychological resilience → parenting style → children’s emotional and behavioral problems" was well supported by the data, with excellent model fit indices (χ²(115) = 212.468, χ²/df = 1.85, SRMR = .070, RMSEA = .067 (90% CI: .052–.080), CFI = .936, TLI = .907). The structural equation modeling diagram is presented below:

b) In the adolescent group, the same model did not meet acceptable fit standards (χ²(115) = 217.915, χ²/df = 1.90, SRMR = .109, RMSEA = .118 (90% CI: .094–.142), CFI = .814, TLI = .733). This is likely attributable to the relatively small effective sample size in the adolescent subgroup (n = 64).

To address your concern more comprehensively and rigorously, we further incorporated the age of children with ASD as a control variable in the full-sample model. The results indicated that the core mediating effect remained significant. Additionally, we have explicitly acknowledged the broad age range of the children as a study limitation in the respective section (see lines 438-441).

Given the absence of substantial differences between fathers and mothers, as well as between boys and girls with ASD, across the study variables, we have chosen to present only the structural equation model for the full sample in the manuscript, omitting separate subgroup analyses.

We sincerely thank you once again for this invaluable suggestion, which has significantly enhanced our exploration and interpretation of the deeper implications underlying the data.

23. Table 1: Educational attainment of whom – I cant find any details in methods.

**Reply:** Thank you for raising this important issue. We sincerely apologize for the lack of clarity in reporting educational attainment information in Table 1. The following revisions and additions have been implemented:

a) The category "Educational attainment" has been disaggregated into two separate items: "Fathers educational attainment" and "Mother's educational attainment," both of which are now reported individually in Table 1;

b) In the "Participants" subsection of the Methods, the educational attainment levels of mothers and fathers are now described separately. The added text is as follows:

"Among the fathers, their educational attainment was distributed as follows: 22 (8.5 %) had completed junior high or below, 49 (19.0 %) senior high, 161 (62.4 %) university, and 26 (10.1 %) held postgraduate degrees (see lines 110-112). Among mothers, 23 (8.9 %) had finished junior high or below, 54 (20.9 %) senior high, 162 (62.8 %) university, and 19 (7.4 %) postgraduate studies (see 114-115 lines)."

c) In subsequent analyses, both fathers and mother's educational attainment were included as separate control variables in the model.

Thank you again for your valuable suggestions, which have significantly strengthened the methodological rigor of this study.

24. Discussion:Remove subsections.

**Reply:** Thank you for your valuable reminder. In the revised manuscript, we have removed all subheadings within the Discussion section and refined the logical flow between paragraphs to ensure a more coherent presentation. We sincerely appreciate your insightful suggestions once again, which have greatly improved the quality of our work.

25. Remove billet points from conclusion.

**Reply:** Thank you for your reminder. In the revised version, we have removed the bullet points and, incorporating suggestions from Reviewer 2, have completely rewritten the Conclusion section. For your convenience during review, the revised conclusion is presented below:

"Current research has clearly delineated the positive role of parental psychological resilience in rehabilitation interventions for children with autism, while also underscoring the pivotal mediating role played by family parenting styles in this process. Thus, enhancing parental psychological resilience and guiding them to adopt adaptive parenting strategies represent a critical pathway for improving socio-emotional development outcomes in children with autism. Future efforts to advance child rehabilitation services, if accompanied by simultaneous psychological support and parenting skills training for caregivers, will help establish synergistic mechanisms, thereby enhancing overall intervention outcomes (see lines 448-456)."

We extend our sincere gratitude for your patient review and guidance, which have significantly enhanced the academic rigor and quality of our manuscript.

**Reviewers' comments:**

**Reviewer #1:**

1. The second paragraph of the introduction emphasizes the roles of independent and dependent variables, but does not mention mediating variables. A clearer explanation of the relationship between the three is needed.

**Reply:** We sincerely appreciate your thorough review and professional guidance. Upon careful re-examination of the manuscript, we acknowledge that the original text did lack clarity in elaborating the relationships between variables.

Following your suggestion, we have reorganized and revised the Introduction section: the second paragraph now introduces "parenting style" as the mediating variable, and the third paragraph systematically elaborates on the concept and classification of parenting styles. This establishes a clear theoretical logic chain of "independent variable - mediating variable - dependent variable." The revised content can be found in lines 75-90.

We extend our thanks once again for your expert advice, which has significantly enhanced the logical rigor and completeness of the Introduction.

2. This study investigates parents of children with autism in the context of Chinese culture. Therefore, in the section on "Parental Psychological Resilience," it is necessary to find some recent studies on the resilience of parents of children with autism in China to better illustrate the value of this study.

**Reply:** We fully agree with your valuable suggestion, which is crucial for improving our Introduction. Accordingly, in the second paragraph, we begin by defining the concept of psychological resilience and reviewing current research by Western scholars, subsequently supplementing it with recent studies focusing on parental resilience in the Chinese context of autism. For your convenience during review, the supplemented content is presented below:

"Research in China indicates that parents of autistic children with higher psychological resilience are better able to positively cope with the multiple challenges in the parenting process, maintain good mental health, and demonstrate more positive parenting functions [11](see lines 66-69)."

Thank you once again for your patient review and valuable guidance.

3. A model diagram of the hypotheses should be included in the "Research Questions and Hypotheses" section (1.3).

**Reply:** Thank you very much for your professional guidance. We have supplemented the "Research Questions and Hypotheses" section with a diagram of our hypothesized model (see line 95) to facilitate reader comprehension of the proposed relationships among variables. For your convenience during review, the hypothesized model diagram is presented below:

4. Is the sample size for this study appropriate? Is this based on similar studies or statistical conclusions?

**Reply:** Thank you for raising this critical question. We fully considered the unique characteristics of our target population (families of children with ASD) and the inherent challenges in data collection. Therefore, during the design phase, we referenced established research practices in this field to determine a feasible sample size. For instance, Hastings et al. (2014) included 110 parents in their study on the psychological mechanisms of parents of children with ASD, while Lin et al. (2023) had a sample size of 203 in a related study. Moreover, recent empirical studies in China within this domain have typically featured sample sizes ranging between 120 and 209 (e.g., Qiu et al., 2021, N=120; Yu, 2017, N=209; Chen et al., 2015, N=152).

These precedents collectively demonstrate that our final sample of 258 valid responses aligns with the research conventions in this field and even represents a relatively larger sample size, which helps enhance the stability of our findings and their practical relevance. We extend our sincere gratitude for your patient review and professional guidance once again.

(Reference 1：Jones, L., Hastings, R. P., Totsika, V., Keane, L., & Rhule, N. (2014). Child Behavior Problems and Parental Well-Being in Families of Children With Autism: The Mediating Role of Mindfulness and Acceptance. American Journal on Intellectual and Developmental Disabilities, 119(2), 171–185. [https://doi.org/10.1352/1944-7558-119.2.171；Reference 2：Lin, Y., Wang, Y., Lin, C., Ni, Q., Jia, R., Chang, Y., & Qi, Y. (2023). The mediating role of perceived social support: alexithymia and parental burnout in parents of children with autism spectrum disorder [Original Research]. Frontiers in Psychology, Volume 14 - 2023.https://www.frontiersin.org/journals/psychology/articles/10.3389/fpsyg.2023.1139618)](https://doi.org/10.1352/1944-7558-119.2.171。）).

5. Please specify whether the parents of children with autism completed the questionnaire voluntarily or in exchange for gifts or compensation.

**Reply:** We sincerely appreciate your careful review. We have supplemented the description of questionnaire administration procedures in the "Methods - Participants and Procedure" section. The specific revisions are as follows:

"All parents voluntarily completed the questionnaires and signed informed consent forms before participation. Upon completion, they received an assessment feedback report which included evaluations of parental psychological resilience, children's emotional and behavioral characteristics, and parenting recommendations(see lines 126-129). "

Thank you again for your valuable suggestions. Your thorough review has directly contributed to improvements in the methodological rigor of our study.

6. At line 298, determine whether χ2/df < 5.

**Reply:** Thank you for your thorough review. Upon further verification, we confirmed that the χ²/df value is 2.17, which is indeed below the threshold of 5. We have supplemented this information in the "3.4 Structural Equation Modeling" subsection of the revised manuscript (lines 232–233), with the specific addition as follows:

"The model demonstrated an acceptable overall fit, and the specific fit indices are presented below: χ²(115) = 249.217, χ²/df = 2.17 < 5, SRMR = .067, RMSEA = .067 (90% CI: .056–.079), CFI = .933, TLI = .904."

Thank you again for your thorough review, which has improved the clarity of our statistical reporting.

7. The discussion section should be a key part of the manuscript. Lines 306–314 should discuss the similarities between the results of this study and previous studies.

**Reply:** Thank you for your valuable suggestions regarding the Discussion section. We fully agree with your perspective and have conducted a thorough review and revision of the relevant section. Specifically, we have made additions at the following locations: the middle (see lines 296–300) and end (see lines 303–305) of the second paragraph, as well as the end of the fourth paragraph (see lines 321–327).

We sincerely appreciate your expert guidance, which has greatly inspired our approach to writing the Discussion and significantly enhanced its depth and academic rigor.

8. In the limitations section, you can explain the large difference in the number of mothers and fathers in the sample size.

**Reply:** Thank you for raising this important issue. We fully acknowledge the notable imbalance in the gender distribution of parent participants in our study (81.4% mothers vs. 18.6% fathers). In accordance with your suggestion, we have added the following discussion in the "Discussion and Limitations" section:

"First, there was a notable imbalance in the gender ratio of parents of children with autism in the sample. This phenomenon may be attributed to the reality that mothers often assume the primary caregiving responsibilities in families with autistic children [56]. Nevertheless, this imbalance might potentially limit the generalizability of the findings to the father population. Future studies should therefore strive for a more balanced gender representation in their samples(see lines 427-432)."

Thank you again for your careful review and effective feedback.

**Reviewer #2:**

The authors' aim was to demonstrate how high parental resilience can improve children's internalizing and externalizing symptoms through the adoption of an authoritative parenting style. The English is fluent and easily understandable. The introduction is well-written and comprehensive. A strengths is the employment of standardized and validated neuropsychological scales.

**Reply:** We are appreciate for your positive feedback on the research objectives, quality of English writing, introduction section, and the standardization of the scales used. In accordance with the suggestions from you and other editorial experts, we have made revisions to the relevant sections of the manuscript.

In my opinion, there are some methodological issues that should be addressed by the authors who request some revisions:

1) METHOD:

The main methodological issues, in my opinion, concern sample selection, inclusion/exclusion criteria, Autism diagnosis, and the assessment of autism level.

**Reply:** We sincerely appreciate your patient review and valuable feedback. In response, we fully concur with your observations and provide point-by-point replies below.

a) Who made the autism diagnosis? (Please specify whether the diagnosis was made in a level II Center, with a medical and psychological team and whether neuropsychometric tests (ADOS-2, ADI) were instrumental or genetic investigations were performed.)

**Reply:** Thank you for raising this important question. Following your suggestion, we have supplemented the diagnostic information for children with autism spectrum disorder (ASD) in the "Methods - Participants" section. For your convenience during review, the added content is presented below:

"All children with ASD were diagnosed by child psychiatrists from tertiary hospitals with extensive experience in autism diagnosis, following the diagnostic criteria outlined in the Diagnostic and Statistical Manual of Mental Disorders, Fourth or Fifth Edition (DSM-IV/DSM-5) (see lines 117-120)."

Furthermore, it should be noted that this study did not employ standardized diagnostic tools such as ADOS-2 or ADI-R, nor were genetic examinations conducted. We acknowledge this as a methodological limitation of our research. Accordingly, we have addressed this issue in the "Research Limitations and Future Directions" section of the manuscript as follows:

"Second, the assessment of children's emotional and behavioral problems primarily relied on questionnaire methods. While this approach offers operational convenience, it is susceptible to biases such as social desirability and recall bias, making it difficult to fully capture the actual symptoms. Future studies should incorporate professional assessment tools (e.g., ADOS-2, VABS) to enhance the objectivity and comprehensiveness of the data(see lines 432-437). "

We extend our sincere gratitude for your valuable guidance again, which has significantly enhanced the rigor and quality of our study. We will give due consideration to this issue in our subsequent research.

b) What criteria were used to make the diagnosis? (Please specify if was based on DSM-5 criteria.)

**Reply:** Thank you for your reminder. The diagnostic criteria used were the clinical standards outlined in the Diagnostic and Statistical Manual of Mental Disorders, Fourth or Fifth Edition (DSM-IV or DSM-5). This information has been added to the Participants section.

c) Given the extreme heterogeneity of autism (now we consider autism spectrum and phenotypes of autism), was an assessment of the autism level performed? (Please specify whether participants were Level I, II, or II; if this information is not available, it should be included in the study limitations, as the level of autism severity could be a factor influencing parenting stress, resilience, and the presence of internalizing and externalizing problems.)

**Reply:** Thank you for raising this important question. We did collect information regarding the severity of autism spectrum disorder. This assessment was primarily based on parent-reported clinical diagnoses from hospitals (specifically, the severity classifications—mild, moderate, or severe—as documented by physicians in medical records). For your convenience during review, the supplemented content is provided below:

"Among the children, 81 (31.4%) were clinically assessed as having mild ASD (including those formerly diagnosed with Asperger's syndrome), 127 (49.2%) as moderate, and 50 (19.4%) as severe (see lines 119-121)."

Secondly, in our statistical analysis, we further included the severity level as a categorical control variable in the model to minimize the potential impact of symptom heterogeneity on the relationships between core variables.

d) What were the exclusion/inclusion criteria? Were neurological and psychiatric comorbidities and relevant clinical conditions excluded? These factors could also influence the above-mentioned aspects.

**Reply:** We sincerely appreciate your valuable suggestions. The inclusion and exclusion criteria for this study were as follows: (1) clinically diagnosed children with autism spectrum disorder; (2) aged 3-18 years; (3) no other serious comorbid conditions reported by parents. The revised content can be found in the Participants section of the Methods. Thank you again for your professional guidance, which provides important implications for refining the methodological description of this study and planning future research directions.

2）Minor issues:

a)- The age range is quite broad. Did the authors perform a data analysis dividing the sample into children and adolescents?This point could be further explored or included as a weakness.

**Reply:** Thank you very much for your important suggestion. Following your recommendation, we first divided the total sample into a child group (3-12 years, n = 194) and an adolescent group (13-18 years, n = 64) for subgroup analysis, in order to test the validity of the theoretical model across different developmental stages. The results are as follows:

In the child group (3-12 years, n = 194), the proposed theoretical model of "psychological resilience → parenting style → children's emotional and behavioral problems" was well supported by the data, with excellent model fit indices (χ²(115) = 212.468, χ²/df = 1.85, SRMR = .070, RMSEA = .067 (90% CI: .052–.080), CFI = .936, TLI = .907). The structural equation modeling diagram is presented below:

In the adolescent group (13-18 years, n=64), the same model did not achieve acceptable fit standards (χ²(115) = 217.915, χ²/df = 1.90, SRMR = .109, RMSEA = .118 (90% CI: .094–.142), CFI = .814, TLI = .733). This outcome may be attributed to the relatively small effective sample size in the adolescent subgroup (n=64).

Second, in accordance with your recommendation, we introduced child age as a covariate in the full-sample model to further verify the robustness of our findings. The analysis demonstrated that the key mediating effect retained its statistical significance after accounting for age, confirming the stability of the proposed mechanism.

Finally, we have added the issue of children's age as a study limitation. For your convenience during review, the supplemented content is provided below: "Finally, although the inclusion of children with autism across a relatively wide age range in this study helps to identify overall trends, it may also obscure differences in emotional and behavioral presentations and family interaction patterns across different developmental stages. Future research could further stratify the sample into developmental stages such as childhood and adolescence, thereby enhancing the study's developmental specificity and interpretive precision (see lines 438-443)."

Thank you again for this insightful suggestion, which has significantly enhanced our data interpretation. We will be sure to consider this issue in subsequent studies.

b)- The questionnaires were completed by 1/5 of fathers and 4/5 of mothers. How do you interpret this data? Is there a difference in resilience, parenting style, and SDQ scores based on the parent completing the questionnaire?

**Reply:** Thank you for your valuable suggestion. Following your recommendation, we conducted independent samples t-tests to examine gender differences across all variables. The complete statistical results have been organized into Table 2 in the "Data Analysis" section (see line 234).

The findings indicate that, among the variables examined, only parental education level showed a statistically significant gender difference. Based on this finding, we included father’s and mother’s education level as control variables in the subsequent structural equation modeling analysis to ensure the accuracy of model estimation.

We sincerely appreciate your professional guidance once again, which has significantly enhanced the rigor and completeness of our data analysis.

c)- Was the parents' social background and educational level analyzed?

**Reply:** Thank you for raising this question. We did collect information on parents' socioeconomic backgrounds in our study, with a particular focus on the key variable of educational attainment. The data analysis for this variable included the following three components:

First, we performed descriptive statistics on parents' educational attainment in the Participants section. For your convenience during review, the supplemented content is provided below:"Among the fathers, their educational attainment was distributed as follows: 22 (8.5 %) had completed junior high or below, 49 (19.0 %) senior high, 161 (62.4 %) university, and 26 (10.1 %) held postgraduate degrees (see lines 110-112). Among mothers, 23 (8.9 %) had finished junior high or below, 54 (20.9 %) senior high, 162 (62.8 %) university, and 19 (7.4 %) postgraduate studies(see lines 114=115)."

Second, we supplemented the results of the correlation analysis of variables in the "Data Analysis" section (see Table 1).

Finally, it was incorporated as a control variable in the structural equation modeling (see Figure 2).

d)- Is there information available indicating whether the parents were receiving parenting support?

**Reply:** Thank you for raising this important question. All families of children with autism spectrum disorder participating in this study were recruited from designated rehabilitation institutions recognized by the China Disabled Persons' Federation. According to publicly available policies in Qingdao City, Shandong Province, general families of children with ASD receiving rehabilitation training in these designated institutions are eligible for a subsidy standard of 2,100 RMB per month per child; for families with financial difficulties (those receiving subsistence allowances or classified as low-income), the subsidy standard is 3,500 RMB per month per child. These specific subsidy standards are detailed in the policy documents of Qingdao City. We appreciate your valuable question once again.

a)- The authors state that greater parental resilience predicts better scores in children with ASD on internalizing and externalizing symptoms, however, this relationship may also be bidirectional. Numerous articles in the literature report higher parental stress in children with ASD and other neurodevelopmental disorders compared to typically developing children, and parental stress may also be linked to child factors such as the severity of autism symptoms, age, and, above all, general adaptive functioning and emotional-behavioral problems (see Operto FF et al. Neuropsychological Profile, Emotional/Behavioral Problems, and Parental Stress in Children with Neurodevelopmental Disorders. Brain Sci. 2021 Apr 30;11(5):584. doi: 10.3390/brainsci11050584; Operto FF et al. Adaptive Behavior, Emotional/Behavioral Problems and Parental Stress in Children With Autism Spectrum Disorder. Front Neurosci. 2021 Nov 25;15:751465. doi: 10.3389/fnins.2021.751465.).

More problematic or more challenging children may place a strain on their parents over time, resulting in increased parenting stress and subsequently lower resilience, with dysfunctional consequences for the entire family well being. Furthermore, we say that parenting stress in chronic situations may increase over time.

This point should be carefully addressed in the discussion.

**Reply:** Thank you for this important suggestion. We fully agree that bidirectional relationships may exist between the variables. We have carefully studied the two recommended articles, which provided substantial insights. These studies clearly indicate that emotional and behavioral symptoms in children with ASD significantly impact parental psychological distress, with stress levels increasing alongside symptom severity, which is consistent with our discussion in the second paragraph of the Introduction. We have added the corresponding citations into the revised manuscript, the content is as follows :

"Thus, even though the emotional and behavioral problems of children with autism place heavy demands on their parents [12,13], parental resilience can still motivate them to adopt more positive and effective strategies when facing parenting challenges(see lines 69-72)."

Thank you again for your valuable input, which has substantially improved the depth and academic rigor of the discussion.

b)- Was a standardized assessment of communication and relational symptoms (e.g., ADOS-2), adaptive functioning (VABS, ABAS, etc.), and emotional-behavioral symptoms conducted? This would be very important; if such data are not available, this point should be included in the study limitations.

Reply: We sincerely appreciate your professional suggestion. Our study has the following limitations in terms of assessment tools: due to the relatively large sample size (N=258) and limited research resources, we were unable to employ standardized behavioral observation and adaptive functioning assessment instruments such as ADOS-2 and VABS. Additionally, some parents of children with autism expressed concerns about in-depth clinical assessments, worrying that these might impose additional burdens on their children, and therefore preferred to participate only in relatively straightforward questionnaire surveys.

Following your recommendation, we have supplemented the "Research Limitations and Future Directions" section with this explanation. For your convenience during review, the added content is presented below:

"Second, the assessment of children's emotional and behavioral problems primarily relied on questionnaire methods. While this approach offers operational convenience, it is susceptible to biases such as social desirability and recall bias, making it difficult to fully capture the actual symptoms. Future studies should incorporate professional assessment tools (e.g., ADOS-2, VABS) to enhance the objectivity and comprehensiveness of the data (see lines 432-437)."

c)- The "Limitations and Future Perspectives" section should be expanded to include the aforementioned limitations of the study. For future perspectives, it would be interesting to propose a longitudinal study on evolutionary trajectories over time.

**Reply:** Thank you very much for your professional guidance. We fully endorse this valuable suggestion you have put forward. In accordance with your recommendations, we have systematically expanded and refined the "Limitations and Future Directions" section of our paper.

Regarding the limitations, we have integrated and explicitly articulated the issues you mentioned above. In the future directions section, following your advice, we have particularly emphasized the design considerations for longitudinal research. For your convenience during review, the supplemented content is presented below:

"Furthermore, there is a need to employ longitudinal research designs to continuously track the dynamic trajectories of parental psychological resilience and child developmental outcomes, and to explore their longitudinal relationships (see lines 443-446)."

Thank you again for your careful review, which has substantially strengthened the scholarly rigor and depth of this research.

d）- In my personal opinion, in my conclusions, I would emphasize the point that rehabilitation therapy performed on the child can benefit from a parallel intervention to support the parent (psychological support, parent training) in order to achieve a synergistic effect and improve family well-being.

**Reply:** We sincerely appreciate your professional suggestion and fully concur with your perspective. Following your recommendation, we have completely rewritten the Conclusion section. For your convenience during review, the revised content is presented below:

"Current research has clearly delineated the positive role of parental psychological resilience in rehabilitation interventions for children with autism, while also underscoring the pivotal mediating role played by family parenting styles in this process. Thus, enhancing parental psychological resilience and guiding them to adopt adaptive parenting strategies represent a critical pathway for improving socio-emotional development outcomes in children with autism. Future efforts to advance child rehabilitation services, if accompanied by simultaneous psychological support and parenting skills training for caregivers, will help establish synergistic mechanisms, thereby enhancing overall intervention outcomes (see lines 448-456)."

Thank you again for your valuable feedback, which has substantially strengthened the practical significance and applicability of our study's conclusions.
