# Supplementary material for: The association between parental resilience and emotional/behavioural problems in children with autism spectrum disorders: The mediating role of parenting style
Source: PLoS One. 2026 Feb 5;21(2):e0329989. doi: 10.1371/journal.pone.0329989 (PMC12875478; doi:10.1371/journal.pone.0329989)
Supplement: Attachment — Submitted filename: Response_to_Reviewers_auresp_2.docx [file pone.0329989.s006.docx]

Response to reviews

Dear Editor,

We now resubmit our manuscript after substantial revision. We thank the reviewers for their excellent comments, which have helped tremendously to improve the quality of the paper. Attached are the point-by-point responses to the reviewer comments and the revised version of our paper.

We hope that the paper is now acceptable for publication. Thanks for considering our paper for publication in your esteemed journal.

Below, reviewer's comments and our responses are presented in **black** and **blue**, respectively.

**Reviewer**

This manuscript examines the association between parental psychological resilience and emotional and behavioral problems in children with Autism Spectrum Disorder (ASD), with parenting style as a mediating mechanism. The topic is relevant, the theoretical framework is appropriate, and the use of validated instruments and structural equation modeling (SEM) is generally sound. The study contributes useful evidence to the literature on family-based approaches to ASD intervention.
However, several issues related to study design, interpretation of findings, figure clarity, and reporting transparency should be addressed before the manuscript can be considered for publication.
**Response:** We are grateful for your encouraging comments and constructive suggestions. Each and every one has been carefully addressed point by point in our revised manuscript.

Major Comments
1. Study design and causal interpretation
The study employs a cross-sectional design, yet causal language is used throughout the manuscript (e.g., “resilience promotes prosocial behavior,” “mitigates emotional symptoms”). While mediation analyses were conducted, such analyses do not establish causal pathways in cross-sectional data.

Recommendation:
Please revise the manuscript to consistently use associational language and clearly state that the mediation findings are statistical rather than causal in nature, particularly in the Abstract, Results, and Discussion sections.

**Response:** We sincerely thank the reviewer for this critical observation. We agree that given the cross-sectional nature of our study, causal language was inappropriate. We have carefully revised the manuscript to use associational language throughout the Abstract, Introduction, and Discussion sections. Furthermore, as recommended, we have explicitly stated in the Discussion section that the mediation findings represent statistical associations rather than confirmed causal pathways, and we have emphasized the need for future longitudinal research to validate these relationships.

2. Common method bias and single-informant data

All key variables (parental resilience, parenting style, and child outcomes) were reported by the same parent respondent. Although Harman’s single-factor test was conducted, this method alone is limited in its ability to rule out common method variance.

Recommendation:
Explicitly acknowledge this limitation and discuss how shared method variance may have influenced the observed relationships. Future research directions should emphasize multi-informant or observational designs.

**Response:** We appreciate the reviewer highlighting this important methodological limitation. We acknowledge that relying exclusively on parent-reported data for both independent and dependent variables introduces the risk of common method variance (CMV), which may inflate the observed relationships, and we agree that Harman’s single-factor test alone is insufficient to completely rule out this bias. In response to your recommendation, we have expanded the "Limitations" section to explicitly discuss the potential influence of shared method variance on our results. Furthermore, we have added a recommendation for future studies to utilize multi-informant designs (e.g., collecting data from teachers or clinicians) and observational measures to enhance the objectivity of the findings.

3. Sample characteristics and generalizability
Participants were recruited from autism intervention institutions in a single province in China, and the majority of respondents were mothers. These factors may limit the generalizability of the findings to other regions, cultural contexts, caregiver roles, or families without access to intervention services.

Recommendation:
Expand the discussion of generalizability limitations and clearly state the population to which the findings are most applicable.

**Response**: We agree with the reviewer that the recruitment of participants from a single province and the predominance of mothers in the sample limit the generalizability of our findings. In accordance with your recommendation, we have expanded the discussion on limitations to explicitly acknowledge these geographical and demographic constraints. We have clarified that the findings may not be fully generalizable to other cultural contexts or to fathers, and we have emphasized the need for future multi-regional studies with more balanced gender representation.

4. Measurement considerations
The permissive parenting subscale demonstrated relatively low internal consistency (α = .67) compared to other measures used in the study.

Recommendation:
Acknowledge this limitation and briefly discuss its potential impact on mediation results involving permissive parenting.

**Response:** Thank you for pointing this out. We acknowledge that the Cronbach’s alpha for the Permissive Parenting subscale (.67) was relatively low compared to the other measures. We have added a statement in the "Limitations" section to explicitly recognize this issue. We also advise readers to interpret the mediation results involving permissive parenting with appropriate caution due to this lower internal consistency.

5. Clarity and quality of Figure 1

Figure 1 (Structural equation model) is difficult to interpret in its current form. The font size, path coefficients, and labels are small and unclear, which makes it challenging to follow the model structure and key findings.

Recommendation:
Please improve the clarity of Figure 1 by:
Increasing resolution and font size
Ensuring all paths and coefficients are clearly legible
Simplifying the figure if possible, or providing a more detailed caption explaining the main paths
Clear and readable figures are essential for accurate interpretation of SEM results.

**Response**:We thank the reviewer for this important feedback regarding the figure's readability.

Clarification on Figure Numbering:We noticed the comment refers to "path coefficients" and the "Structural Equation Model." In our manuscript, Figure 1 is the conceptual Assumptions Model (theoretical framework without coefficients), while Figure 2 displays the SEM results with specific path coefficients. We assume the reviewer intended to critique the clarity of Figure 2 (the Results Model).

Improvements Made:We have completely processed Figure 2 to address the visibility issues:

1. Enlarged Typography: We have significantly increased the font size of all path coefficients and variable labels to ensure they are clearly legible.
2. High-Resolution: The figure has been replaced with a high-resolution version to eliminate blurriness.
3. Visual Clarity regarding Simplification: Regarding the suggestion to simplify: We carefully considered removing the control variables from the figure. However, we decided to retain them to ensure the transparency and completeness of the reported statistical model, allowing readers to see the exact estimated paths. Instead of removing them, we improved the layout and line contrast to minimize visual clutter while keeping the model specification precise.

The revised Figure 2 is now presented in the revised manuscript.

6. Covariates and model specification
Parental age and child age were included as covariates for selected outcomes; however, the rationale for their inclusion is not fully explained, nor is it clear why other demographic variables (e.g., parental education) were not controlled.

Recommendation:
Please clarify the theoretical or empirical rationale for the selected covariates and explain the decision-making process for including or excluding other demographic variables in the SEM.

**Response:** We appreciate the reviewer’s attention to model specification. We wish to clarify our variable selection process. In our preliminary analysis, we screened all collected demographic variables as potential control variables. To maintain model parsimony, demographic variables that showed no significant associations with the outcome variables were excluded from the final Structural Equation Model. However, we explicitly retained parental education levels, child age, and autism severity as control variables in the final model (as noted in Section 3.4), because they showed significant effects. Regarding the reviewer's concern about "parental education": it was indeed controlled for in the final model. We retained these specific variables because existing literature consistently shows that child developmental stage and symptom severity are fundamental predictors of behavioral problems, while parental education (as a proxy for SES) is a stable predictor of parenting styles. We have added a sentence in Section 3.4 to explicitly state this selection rationale.

7. Missing data handling
The manuscript states that questionnaires with “substantial missing data” were excluded, but no criteria are provided, and the handling of remaining missing data is not described.

Recommendation:
Please clarify:
The criteria used to define “substantial” missing data
Whether any missing values remained in the final dataset
The estimation method used in Mplus for handling missing data (e.g., FIML)

**Response:** We apologize for the lack of clarity regarding data screening. In this study, questionnaires with incomplete responses (often due to participants declining to answer specific privacy-related items) were strictly excluded from the dataset (listwise deletion) to ensure data quality. Consequently, the final sample of 258 participants contained no missing data. We have revised Section 2.1 to clarify this exclusion criterion and the completeness of the final dataset.

8. Interpretation of effect sizes
The Results and Discussion sections focus primarily on statistical significance, with limited discussion of the magnitude or practical significance of the observed effects.
Recommendation:
Please include a brief discussion of the practical or clinical relevance of the effect sizes, particularly in relation to family-based interventions for children with ASD.

**Response:** Thank you for this valuable suggestion. We agree that statistical significance should be contextualized with practical relevance. We have added a paragraph in the Discussion section to interpret the effect sizes. We argue that while the standardized coefficients are small-to-medium, they are clinically meaningful because parenting style is a modifiable factor. Identifying such malleable targets is crucial for family-based interventions, where even incremental improvements can significantly enhance the quality of life for families of children with ASD.

Minor Comments
Terminology consistency:
Terms such as parental psychological resilience, parental resilience, and family resilience are used interchangeably. Please define these constructs clearly and use consistent terminology throughout the manuscript.

**Response**: We apologize for the inconsistent terminology. We have carefully reviewed the manuscript and standardized the terminology. We now use "Parental Psychological Resilience" consistently throughout the text to refer to the construct. Occurrences of other variations have been corrected.

Residual correlations:
Provide a clearer justification for correlating residuals between permissive and authoritarian parenting beyond reliance on modification indices.

**Response:** Thank you for requesting a clearer justification. We allowed the residuals of permissive and authoritarian parenting to correlate not merely based on modification indices, but on theoretical grounds. Both styles represent maladaptive approaches characterized by a lack of constructive structure or emotional support. Theoretically, this specification was justified because both styles represent maladaptive parenting strategies that often co-occur in high-stress caregiving contexts. According to Coercion Theory [1], parents overwhelmed by challenging behaviors may vacillate between harsh control (authoritarian) and submission to child demands (permissive) in an effort to manage distress, resulting in a significant positive correlation between these two dimensions.We have added this theoretical justification to Section 3.4.

[1]Patterson, G. R. (1982). Coercive family process. Eugene, OR: Castalia.
